# Backwash sediment record of the 2009 South Pacific Tsunami and 1960 Great Chilean Earthquake Tsunami

**DOI:** 10.1038/s41598-020-60746-4

**Published:** 2020-03-05

**Authors:** Brieuc Riou, Eric Chaumillon, Catherine Chagué, Pierre Sabatier, Jean-Luc Schneider, John-Patrick Walsh, Atun Zawadzki, Daniela Fierro

**Affiliations:** 1 LIENSs UMR 7266-CNRS, Université de La Rochelle, F-17000 La Rochelle CEDEX, France; 2 EPOC UMR 5805-CNRS, Université de Bordeaux, F-33615 Pessac CEDEX, France; 3 School of Biological, Earth and Environmental Sciences, UNSW, Sydney, 2052 NSW Australia; 4 EDYTEM UMR 5204-CNRS, Université Grenoble Alpes, Université Savoie Mont Blanc, F-73000 Chambéry CEDEX, France; 5 University of Rhodes Island, Narragansett, RI 02882 USA; 6 Australian Nuclear Science and Technology Organisation, Lucas Heights, 2234 NSW Australia

**Keywords:** Natural hazards, Ocean sciences

## Abstract

Following recent tsunamis, most studies have focused on the onshore deposits, while the offshore backwash deposits, crucial for a better understanding of the hydrodynamic processes during such events and offering an opportunity for sedimentary archives of past tsunamis, have mostly been omitted. Here, we present a unique sedimentary record of the backwash from two historical tsunamis sampled in a sheltered bay in American Samoa, namely the 2009 South Pacific Tsunami and the 1960 Great Chilean Earthquake Tsunami. Although not always concomitant with a marked grain size change, backwash deposits are identified by terrestrial geochemical and mineralogical signatures, associated with basal soft sediment micro-deformations. These micro-deformations, including asymmetric flame structures, are described for the first time in historic shallow marine backwash deposits and lead us to propose an improved depositional mechanism for tsunami backflow based on hyperpycnal currents. Moreover, this study brings a potential new criterion to the proxy toolkit for identifying tsunami backwash deposits, namely the basal soft sediment micro-deformations. We suggest that further studies focus on these micro-deformations in order to test the representability of this criterion for tsunami backwash deposits. Sheltered shallow marine environments in areas repeatedly impacted by tsunamis have a higher potential for the reconstruction of paleo-tsunami catalogs and should be preferentially investigated for coastal risk assessment.

## Introduction

Over the last two decades, interest in tsunami-related research has increased significantly, with peaks in the number of published articles following the 2004 Indian Ocean Tsunami (IOT), the 2009 South Pacific Tsunami (SPT) and the 2011 Tohoku-Oki Tsunami (TOT)^1^. However, most studies have focused on onshore deposits, with only a few tackling the issue of backwash depositional processes^2–16^. Unlike onshore deposits, offshore deposits are not subject to subaerial erosion and less to anthropic reworking, but can be altered by waves, currents, mixing and bioturbation^1,16^. Most of the studies of historic tsunami backwash were carried out in open beach environments following the 2004 IOT and 2011 TOT^5,13,14,16–18^. However, such environments have a poor preservation potential due to their exposure to waves. In contrast, sheltered bays may provide a higher preservation potential due to less reworking by waves. Thus, the choice of the study zone is key when looking for marine backwash deposits. We suggest that in shallow marine sheltered environments characterized by a low hydrodynamic setting, a complete and uninterrupted record is more likely to be preserved.

Most studies of backwash deposits are based on grain size, geochemical data and microfossils^1^. Shallow marine tsunami deposits are usually characterized by an increase of the mean grain size within usually fine marine mud, due to the inclusion of coarse terrestrial sediment originating from the onshore-inundated or beach areas^5,6,12,15,16^, often accompanied by a higher Ti/Ca ratio reflecting the terrestrial input^8,15,16^. Deep offshore foraminifera species, dragged from the oceanic floor to the shallow coastal zones by the tsunami wave, can also often be found in shallow marine backwash deposits^4,6,19,20^.

**Figure 1:**
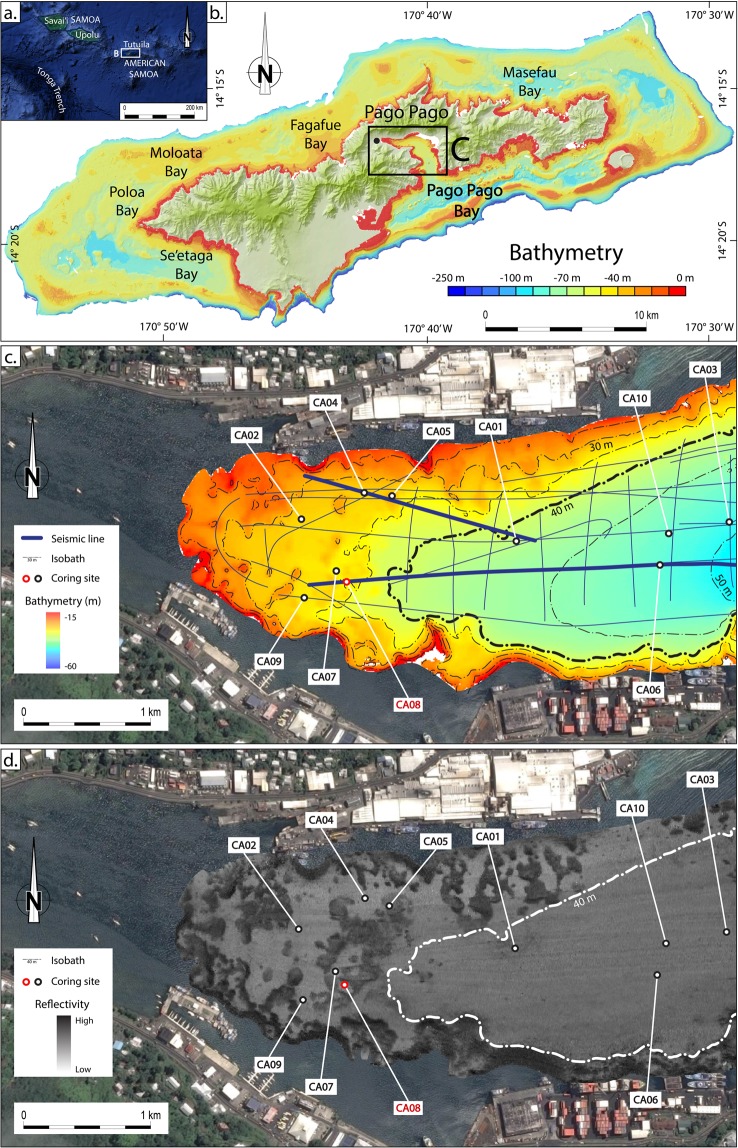
(**a**) Location of American Samoa and Tutuila in the southwest Pacific Ocean (modified from Google, Maxar Technologies). (**b**) Map of Tutuila with bathymetric data (modified from NOAA, 2018; https://data.noaa.gov/dataset/dataset/gridded-bathymetry-of-tutuila-island-american-samoa-south-pacific – acknowledgments to NOAA Coral Reef Ecosystem Division, Pacific Islands Fisheries Science Center and the Pacific Islands Benthic Habitat Mapping Center, School of Ocean and Earth Science and Technology, University of Hawaii). (**c**,**d**) Map of Pago Pago Bay with 1 m high resolution processed bathymetric data (processed using CARAIBES software, IFREMER) and seafloor reflectivity data (processed using SonarScope software, IFREMER), respectively, acquired during the SAMOA-SPT campaign (5 m isobaths) and location of the ten cores.

Tutuila Island (American Samoa) is a volcanic island located in the southwest Pacific less than 200 km from the northern end of the Tonga Trench (Fig. 1a,b). Previous work suggests that Pago Pago Bay has been subject to at least two 1000-year phases of frequent tsunami occurrence during the late Holocene^21^. More recently, the island has been hit by several destructive tsunamis generated all around the Pacific Ocean, including the 2009 SPT and the 1960 Great Chilean Earthquake Tsunami (GCET)^22,23^. Tutuila has a very indented coastline, with deep and narrow bays (Fig. 1b). Owing to its very calm hydrodynamic conditions and the destructive impact of the 2009 SPT, the deepest and most sheltered bay, Pago Pago Bay (Fig. 1), was chosen for this study because it is most likely to provide an ideal setting for the preservation of recent shallow marine tsunami deposits.

## Study Area and Setting

Tutuila Island is part of the Samoan archipelago, a 500 km long volcanic hotspot trail (Fig. 1a,b). Savai’i, which is the oldest island in the west of the volcanic trail, is approximately 5 Ma old^24^, while Tutuila’s shield-building volcanism started 1.5 Ma ago. The island is composed of five volcanoes, with the Pago volcano being the most active^25^. The dominant volcanic rocks are mainly alkaline olivine basalts that make up the calderas, overlain by basanitoids, basanites and olivine nephelinites emplaced after the erosion and collapse of the calderas. All these rocks are characterized by a high titanium content^26^.

The main bay of the island, Pago Pago Bay (Fig. 1c,d), was formed by the inundation of the Pago caldera due to post-volcanic subsidence and erosion^27^. It is a long (5 km), deep (10 to 60 m) and narrow (<1 km) bay ending in an amphitheater head characterized by steep slopes and a reduced coastal plain. The inner part of the bay is completely sheltered from ocean and storm waves, even during the most powerful cyclones that have been reported, and is home to Pago Pago Harbor. The only waves able to reach the inner part of the bay and impact seafloor sedimentation are tsunami waves, as reported during recent events.

Over the last century, more than 100 minor tsunamis have reportedly hit Pago Pago Bay^22,23^. However, three tsunamis stand out. The first one is the 1917 tsunami generated by an earthquake in the northern end of the Tonga Trench. The first observed wave reached 3 m at the head of the bay, causing infrastructure damage, including houses and a church, but no casualties^22^. The 1960 GCET was recorded with a first wave reaching up to 3.5 m, causing little damage and no casualties^22^. The latest was the 2009 SPT generated in the same area as the 1917 tsunami^23,28,29^, with a first wave reaching up to 7 m^30^. It was the most destructive historical tsunami recorded in Pago Pago Bay; it caused considerable damage in the bay up to 500 m inland and 34 deaths around the island^31,32^.

During the same period, cyclones have reached American Samoa nearly every year, with two severe cyclones standing out: Cyclone Ofa in 1990 and Cyclone Val in 1991. The former passed 160 km west of Tutuila and caused heavy rain and flooding with wind gust up to 170 km/h^33^. The latter, said to be the strongest and most destructive cyclone since 1889, passed right over Tutuila with winds reaching 185 km/h, causing heavy rain and flooding^34^. A few months after the 2009 SPT, Tropical Cyclone René made landfall in American Samoa (11–13 February 2010)^35,36^, while nearly a year later, between 22–24 January 2011, Tropical Cyclone Wilma hit American Samoa, with 243 mm rainfall recorded^37^. Such cyclones may cause flashfloods due to heavy rain, resulting in substantial run-off in the bay. However, no large waves were recorded in Pago Pago Bay during these cyclones.

## Results

### Geophysical analysis

The geomorphological characteristics observed from the bathymetry of the Pago Pago Bay seafloor has already been studied and discussed by Riou *et al*.^21^. Based on the bathymetry and the reflectivity surveys, two geomorphologic domains were identified (Fig. 1c,d). The outer domain is characterized by a smooth topography and a steady and gentle 0.5° slope, and extends seaward from a slope break (10 to 15° slope) between the 35 and 40 m isobaths. It displays a homogenous medium reflectivity. The inner domain, which extends between the slope break and the coastline, is characterized by an alternation of mounds and troughs. The mounds have a roughly round shape and are a few meters high (<5 m) by a few tens of meters wide (<50 m), delimitating more or less connected troughs. The troughs display low reflectivity while the mounds display very high reflectivity. Ten shallow sediment cores were collected both in the troughs and on the mounds. Sediments are very homogenous and are dominated by medium to coarse silt (77.5 to 88.5% mud, 11.5 to 22.5% sand). Consequently, the variations in seafloor reflectivity are most likely to be due to slope changes on the flanks of the mounds.

**Figure 2:**
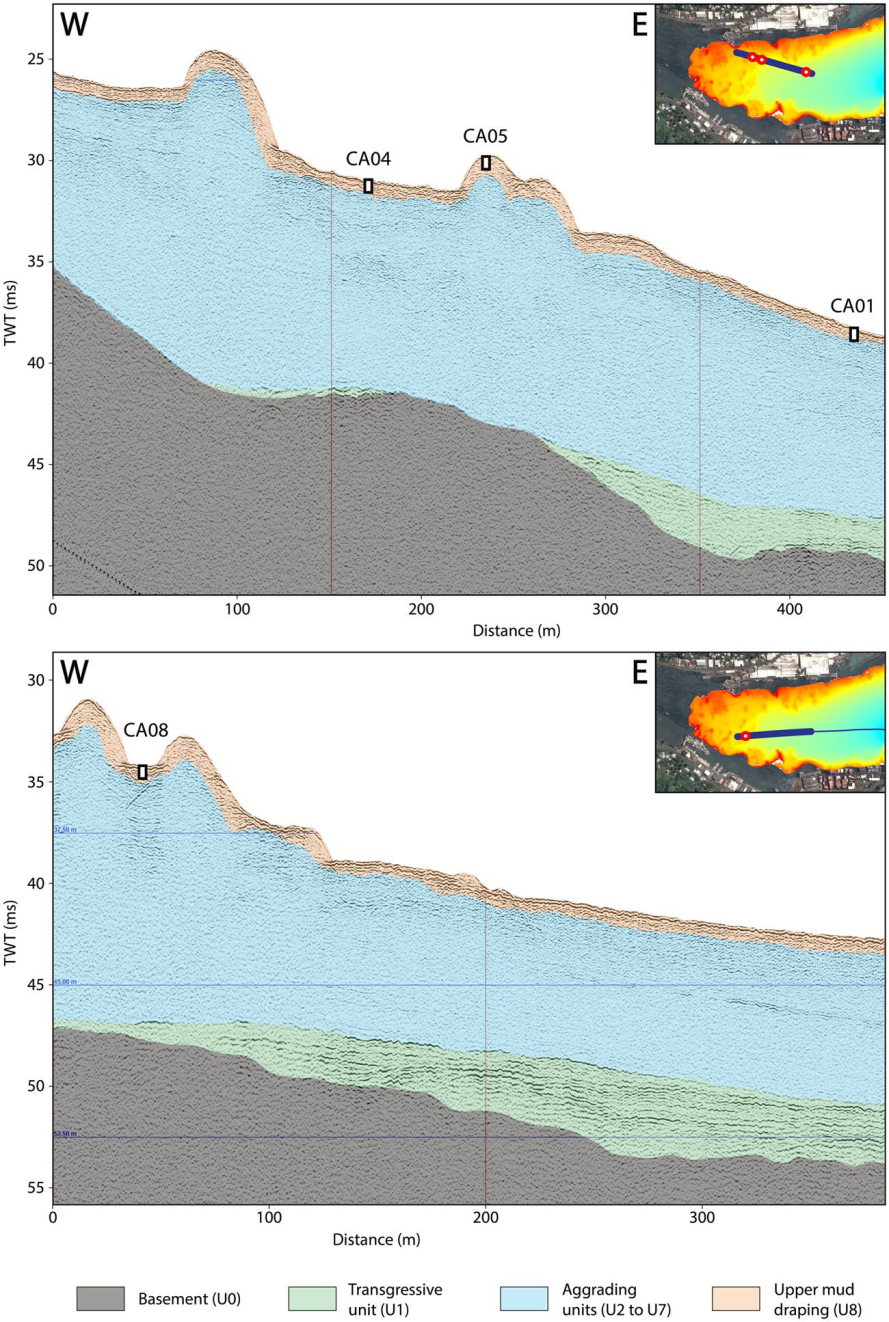
Interpreted west-east seismic profiles Pago-27 (top) and Pago-22 (bottom) processed using Delph seismic interpretation software (iXBlue), simplified according to the interpretation and nomenclature given by Riou *et al*.^21^, with the location of cores CA04, CA05, CA01 and CA08 on the profiles.

Nine sediment sub-units (U0 to U8) have been identified on seismic profiles Pago-27 and Pago-22 by Riou *et al*.^21^. For the purpose of this study, they have been grouped in four sets of units (Fig. 2): (1) the volcanic basement (U0), (2) the transgressive unit (U1), (3) the aggrading units (U2 to U7) and the upper draping unit (U8). The basement is characterized by a transparent seismic facies and limited at the top by a major erosional surface (EU0). The transgressive unit is characterized by retrograding landward-oriented onlaps, while the aggrading units consist of an alternation of muddy and coarse coral debris units pinching-out seaward^21^. The upper draping unit composed of mud is characterized by sub-horizontal and sub-parallel continuous reflectors.

### Grain size analysis

**Figure 3:**
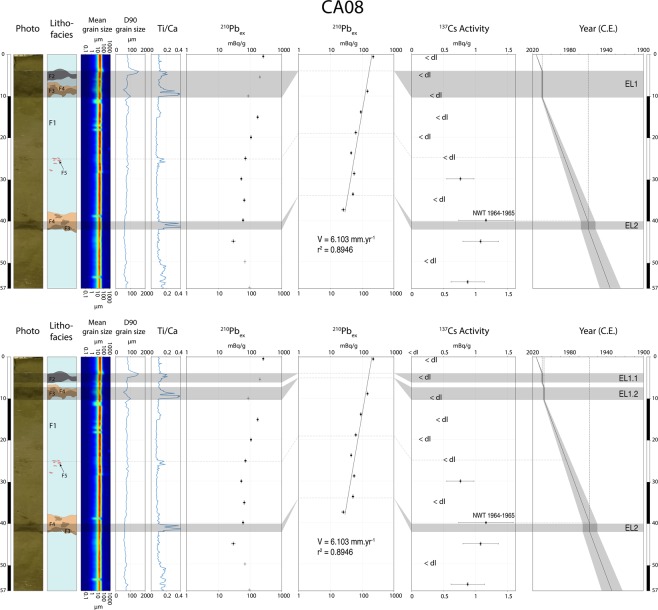
Multi-proxy analysis of core CA08; the top panel illustrates the first hypothesis with two event layers, the lower panel illustrates the second hypothesis with distinction of three event layers; from left to right, respectively, photograph, lithofacies log, grain size (mean and D90), Ti/Ca ratio, excess ^210^Pb excess activities (raw and corrected), ^137^Cs activities and age model. Grey shaded bars represent the 2009 South Pacific Tsunami and the 1960 Great Chilean Earthquake Tsunami event layers. For ^137^Cs activities, <dl were used to plot values below the detection limit. NWT: Nuclear Weapon Tests.

Cores described in this study were sampled in the upper draping unit (U8) in both the inner and outer domains (Fig. 1c,d). Visually, the sediment sequences are mainly homogenous except for a few discontinuous layers of slightly darker sediment. These are mostly found in the first ten centimeters, although some occur deeper in some cores. No major erosional surfaces are observed in the cores. Results of laser particle size analysis do not show major grain size variations along the cores, which are composed of seemingly homogenous silt. Only a discrete increase of the coarser fraction (D90) is observed at 5 cm depth in core CA08 (Fig. 3).

### Geochemical analysis

**Figure 4:**
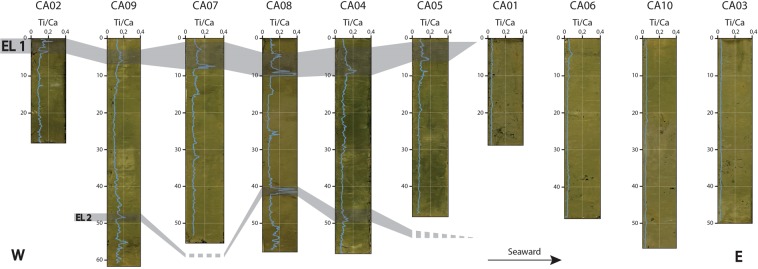
Correlation of all ten cores projected along a west (left) to east (right) transect (see Fig. 1), with photograph and Ti/Ca ratio for each core. Grey shaded bars represent the 2009 South Pacific Tsunami event layer (EL1) and 1960 Great Chilean Earthquake Tsunami event layer (EL2).

**Figure 5:**
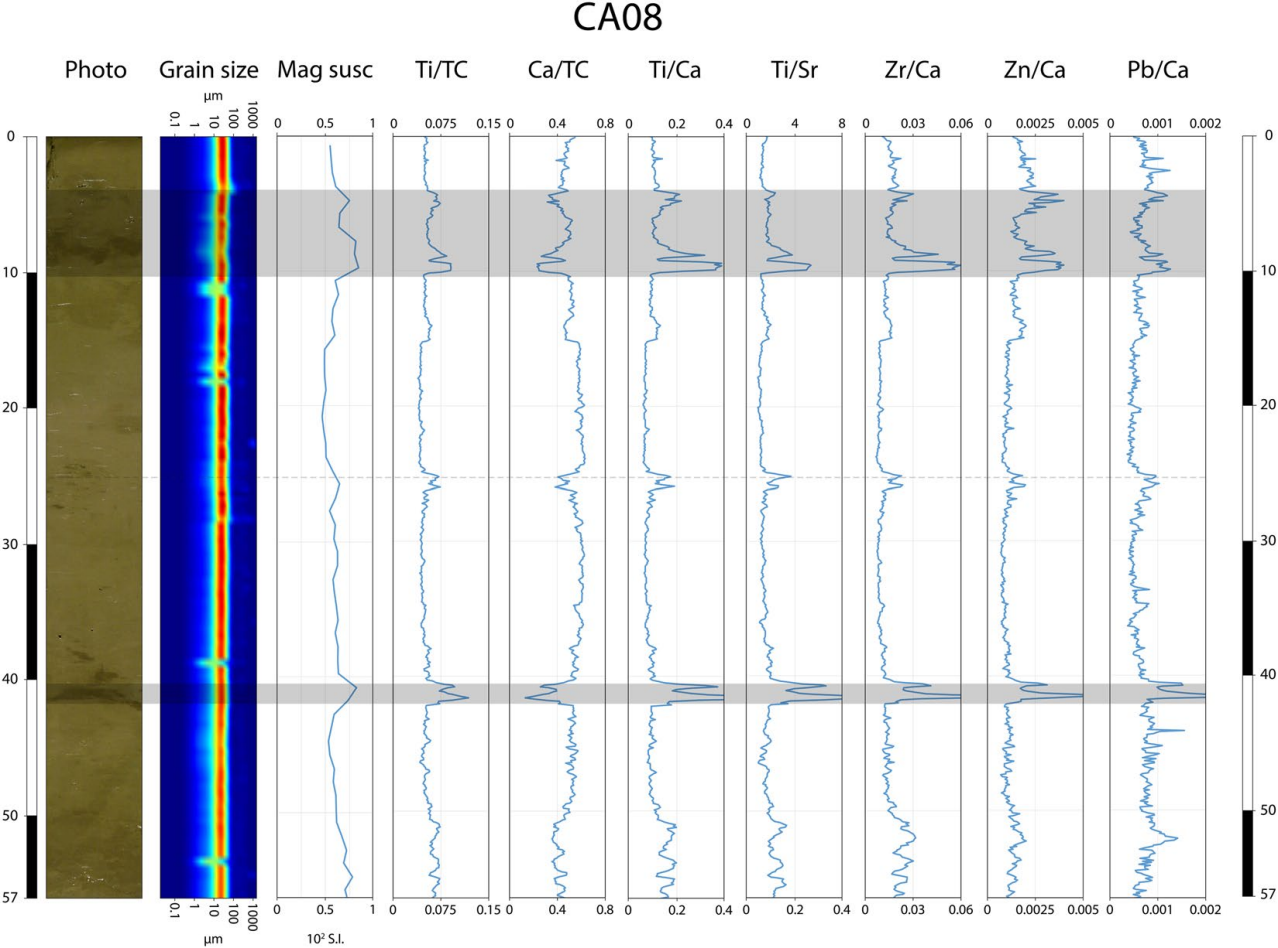
Multi-proxy analysis of core CA08; from left to right, photograph, grain size, magnetic susceptibility and XRF geochemical data (Ti/TC, Ca/TC, Ti/Ca, Ti/Sr, Zr/Ca, Zn/Ca, Pb/Ca). For all element ratios, high values represent an increase in the terrestrial input. Ti and Ca are normalized over the total counts (TC). Grey shaded bars represent the 2009 South Pacific Tsunami event layer (top) and the 1960 Great Chilean Earthquake Tsunami event layer (bottom).

XRF analyses reveal pronounced geochemical variations in a number of cores, as shown by the Ti/Ca ratio (Fig. 4). In core CA08, four main Ti/Ca peaks are observed (Fig. 3): a first small peak at 4–6 cm depth, a second well- marked peak at 8–10 cm depth, a third small peak at 24–26 cm depth and a fourth very high peak at 40–42 cm depth. These Ti/Ca peaks occur in the discontinuous dark sediment layers seen in the core section. Similar peaks are observed in the first 10 cm of all proximal cores (0–5 cm, CA02; 3–7 cm, CA09; 2–8 cm, CA07; 4–10 cm, CA08; 3–10 cm, CA04; 2–6 cm CA05, Fig. 4). In addition, a second deeper peak is found in cores CA04 and CA09, at 48–50 cm and 47–49 cm depth, respectively (Fig. 4). In all cores, normalized Ti and Ca profiles are negatively correlated. Each Ti/Ca peak is due to an increase of Ti and a decrease of Ca, as shown for core CA08 (Fig. 5). Similar trends are observed for the Ti/Sr, Zr/Ca, and Zn/Ca ratios. For the Pb/Ca ratio, the same four peaks are found, but several additional smaller and thinner peaks are also observed. Magnetic susceptibility obtained for core CA08 displays three peaks at 6 cm, 8 to 10 cm and 41 cm depth, correlated with the Ti/Ca peaks (Fig. 5).

### Thin section analysis

**Figure 6:**
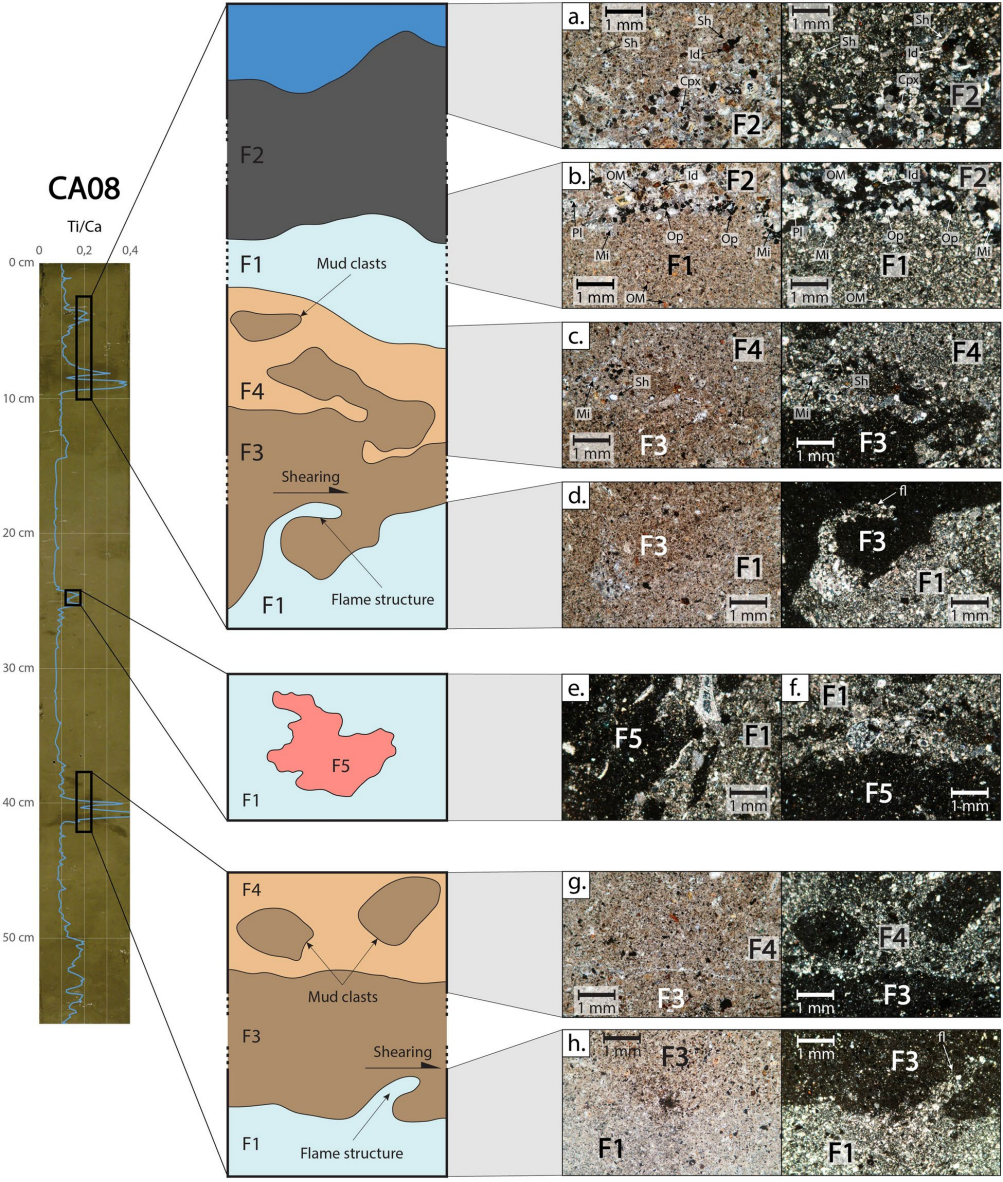
Photograph and Ti/Ca profile for core CA08, with interpretative sections of interest from the core, based on photographs of thin sections, and their position in the core. Note that photographs a., b., c., d., g. and h. are shown twice, once in analyzed light (left) and once in analyzed polarized light (right). Note that photographs a., c. and g. represent the top of each interval with high Ti/Ca, while photographs b., d. and h. represent their base. F1: homogenous silt, micritic mud and aggregates, shells, organic matter (OM). F2: normally graded very fine to fine sand, micritic aggregates, clay aggregates rich in organic matter (OM), opaque minerals (Op), shells (Sh), plant debris, iddingsite (Id), clinopyroxene (Cpx) and plagioclase (Pl). F3: non-graded terrigenous clay with asymmetric flame structures (fl) and rip-up clasts at the base. F4: normally graded clayey silt, mix of terrigenous clay (F3) and micritic mud and aggregates (F1) with shells (Sh).

Five sediment facies (labelled F1, F2, F3, F4 and F5; Fig. 6) were identified based on the grain size and mineralogy observations of thin sections from core CA08,. Facies F1 corresponds to homogenous silt and exhibits a low and constant Ti/Ca ratio throughout most of the core. It is characterized, in order of abundance, by micritic mud and aggregates, bioclastic shells (bivalves, gastropods, spicules), clay aggregates with a high organic matter content, and plant debris (Fig. 6b).

Facies F2 is a normally graded detritic fine to very fine sand that corresponds to a short maximum in Ti/Ca at 4–6 cm depth in core CA08. It is characterized, in order of abundance, by micritic aggregates, rounded clay aggregates with high terrestrial organic content including vascular plant debris (leaves, stems, roots), bioclastic shells (bivalves, gastropods, spicules), along with rounded 200–500 µm iddingsite (altered olivine crystals), clinopyroxene (augite) and plagioclase (labradorite) minerals. In addition, opaque minerals, possibly magnetite, are found at the base of the layer. This facies shows a sharp basal contact and a graded upper contact (Fig. 6a,b).

Facies F3 is a detritic clay exhibiting high Ti/Ca, found at 8–10 cm and 40–42 cm depth. It is characterized by homogenous clay and the absence of carbonated material (Fig. 6c,f). It includes various small volcanic mineral fragments (<100 μm) such as iddingsite, clinopyroxene and plagioclase. It stands out from the background by its darkness in analyzed polarized light. It displays sediment deformation, with a sharp basal contact. Micro-deformations are asymmetric flame structures (Fig. 6d,f), evolving occasionally into rip-up clasts composed of F1 material. Even though cores are not oriented, asymmetric flame structures are all oriented in the same direction. These rip-up clasts are found mostly at the base of the layer and display no mixing with the surrounding F3 sediment.

Facies F4 is a transitional normally graded clayey silt facies exhibiting decreasing upward Ti/Ca, found at 7–8 cm and 38–40 cm depth. It consists of a clayey to silty matrix composed of a mix of F1 and F3, with F3 mud clasts at the base (<2 mm, Fig. 6c,e).

Facies F5 (24–26 cm, CA08) is similar to F3 in terms of mineralogy and grain size, but does not feature any micro-deformations. It is only found as small dispersed clasts (<5 mm).

Smear samples from Laolao and Pago streams onshore are composed, in order of abundance, by plant debris and iddingsite, augite and labradorite minerals. Smear samples from the beach are characterized mainly, in order of abundance, by sub-rounded micritic aggregates, bioclastic shells and plant debris.

### ^210^Pb and ^137^Cs dating

The ^210^Pb excess activity downcore profile shows a regular decrease punctuated by a distinct drop at 10 cm depth (Fig. 3). These low ^210^Pb_ex_ activities correspond to facies F3 layers. Because they are considered instantaneous deposits, facies F2, F3 and F4 are excluded for the construction of an event-free sedimentary record, following^38^ Arnaud *et al*. In the case of the F1 layer observed at 6–7 cm depth, two hypotheses are formulated (see below). In the first hypothesis, the F1 layer altogether with the F2 and F3 layers just above and below, respectively, is considered as part of an instantaneous event and is also excluded for the elaboration of an event-free sedimentary record. In the second hypothesis, the F1 layer is not considered as part of an instantaneous event and is considered in the construction of an alternative sedimentary record. Facies F5 is found highly dispersed and discontinuous in the core and thus was not subtracted. Corrected ^210^Pb_ex_ activities obtained by subtraction of the event layers are plotted on a logarithmic scale and reveal a linear trend, inferring a mean sedimentation rate of 6.1 ± 0.2 mm.yr^−1^. In addition, values below 45 cm depth were not taken into account for estimation of the sediment rate because they are not considered representative of the upper 40 cm. Ages were calculated using the CFCS model (Constant Flux Constant Sedimentation)^39^ applied to the original sediment sequence to provide a continuous age-depth relationship (Fig. 3), and this age-to-depth model was then corroborated with the ^137^Cs activities (Fig. 3). While most samples measured were below or close to the detection limit, the ^137^Cs maximum activity occurs at 40 cm depth. It is commonly accepted that this peak corresponds to 1964–1965 in the Southern Hemisphere, following the Test Ban Treaty in 1962^40,41^. The shallower ^137^Cs peak (30 cm) can be attributed to late stratospheric fallouts^40^, while the deeper peak (55 cm) is most probably due to downward diffusion of ^137^Cs along the sediment column^42^. The good agreement between the ages derived from the ^210^Pb_ex_-CFCS model and the ^137^Cs peak provides a well-constrained continuous age-to-depth relationship.

## Implications

At first sight, the homogenous visual aspect of all cores, except for the slight change of color, and the lack of major visible grain size variations suggest that the superficial sediment fill of Pago Pago Bay could be interpreted as homogenous silty sediment settling in a calm hydrodynamic environment. However, XRF data and microscopic observations of thin sections reveal the presence of irregular and discontinuous thin layers. These layers have a different geochemical signature, mineralogical assemblage, but also grain size not always detected by the laser particle size analyzer. The only discernable change was an increase of the coarser fraction at 5 cm depth in core CA08. The higher Ti/Ca ratio most likely reflects an increase in the terrestrial input, as also observed elsewhere^8,15,16^. Indeed, titanium originates mostly from land and is a component of several volcanic rocks forming Tutuila, such as basanitoids^26^. On the other hand, the main source of calcium is most likely to be from marine carbonates^1,43^. This trend is supported by several other element ratios, such as Ti/Sr, Zr/Ca, Zn/Ca, Pb/Ca and Mn/Ca (Fig. 5). As for calcium, strontium is mainly found in marine carbonates, while zirconium, like titanium, is naturally present in the volcanic rocks of Tutuila. Zinc and lead are most probably sourced from industrial activities in Pago Pago and its harbor, such as the tuna cannery. The terrestrial origin of the high Ti/Ca intervals is confirmed by the mineralogical composition of the layer at 4–6 cm depth in core CA08. Pago Pago Bay has no major fluvial tributary, meaning that the background sedimentation is mostly marine-influenced and dominated by carbonated micritic sediment and shells, as described for F1, and reported by Morrison *et al*.^44^. F2 is also dominated by carbonated micritic sediment and shells but, unlike F1, shows a significant proportion of volcanic minerals (15–20%). These minerals match those observed in the smear samples from Laolao and Pago streams and correspond to those described for the Pago volcanic rocks by Macdonald (1967)^45^. This confirms that F2 records an increase of terrestrial input, with sediment sourced from the erosion of the onshore volcanic shield. Moreover, this layer is coarser than the background sedimentation. This attests of a high energy event, which is coherent with stronger erosion of the volcanic soil onshore.

Layers at 8–10 cm and 40–42 cm depth in core CA08 are composed of F3 and show no change in grain size. There, only the higher than normal Ti/Ca ratio suggests a higher terrestrial input. However, these layers display a very sharp basal contact with the underlying deformed sediments. The layer boundaries show asymmetric flame structures with rip-up clasts (F1, buckling) at the base of both layer^46,47^. These observed structures indicate syn-depositional reworking with shearing of superficial soft and cohesive sediment beneath a dense and cohesive gravity flow^48,49^. Indeed, the lack of mixing between the soft superficial sediment (F1) and the upper layer (F3) implies that the upper sediment layer was highly cohesive. Similar deformation structures can be found at the base of hyperpycnal flows such as turbidites, when the underlying superficial sediment is very fine and cohesive^49,50^. The higher terrestrial signature of these layers suggests a strong land-to-sea hyperpycnal flow coming from the inundated area into the bay. Given the context of Pago Pago Bay, two types of events could generate backflows with such impact on the seafloor: flashfloods due to heavy rain during a severe cyclone, or a strong tsunami backwash.

Based on these interpretations, two hypotheses were formulated regarding the number of event layers observed in core CA08. In the first hypothesis, two event layers are considered (Fig. 3a). The first and shallowest event layer (EL1, Fig. 3a and 6) at 4–10 cm depth is characterized by the succession of facies F3 (8–10 cm), facies F4 (7–8 cm), facies F1 as remobilized marine background sediment (6–7 cm) and facies F2 (4–6 cm). The second and deepest event layer (EL2, Fig. 3a and 6) at 38–42 cm depth is composed of facies F3 (40–42 cm) overlain by facies F4 (38–40 cm). In the second hypothesis, three event layers are considered (Fig. 3b). The first and shallowest event layer (EL1.1, Fig. 3b and 6) at 4–6 cm depth is composed of facies F2. The second event layer (EL1.2, Fig. 3b and 6) at 7–10 cm depth is composed of facies F3 (8–10 cm) overlain by facies F4 (7–8 cm). The third and deepest event layer (EL2, Fig. 3b and 6) at 38–42 cm depth is composed by facies F3 (40–42 cm) overlain by facies F4 (38–40 cm).

**Figure 7:**
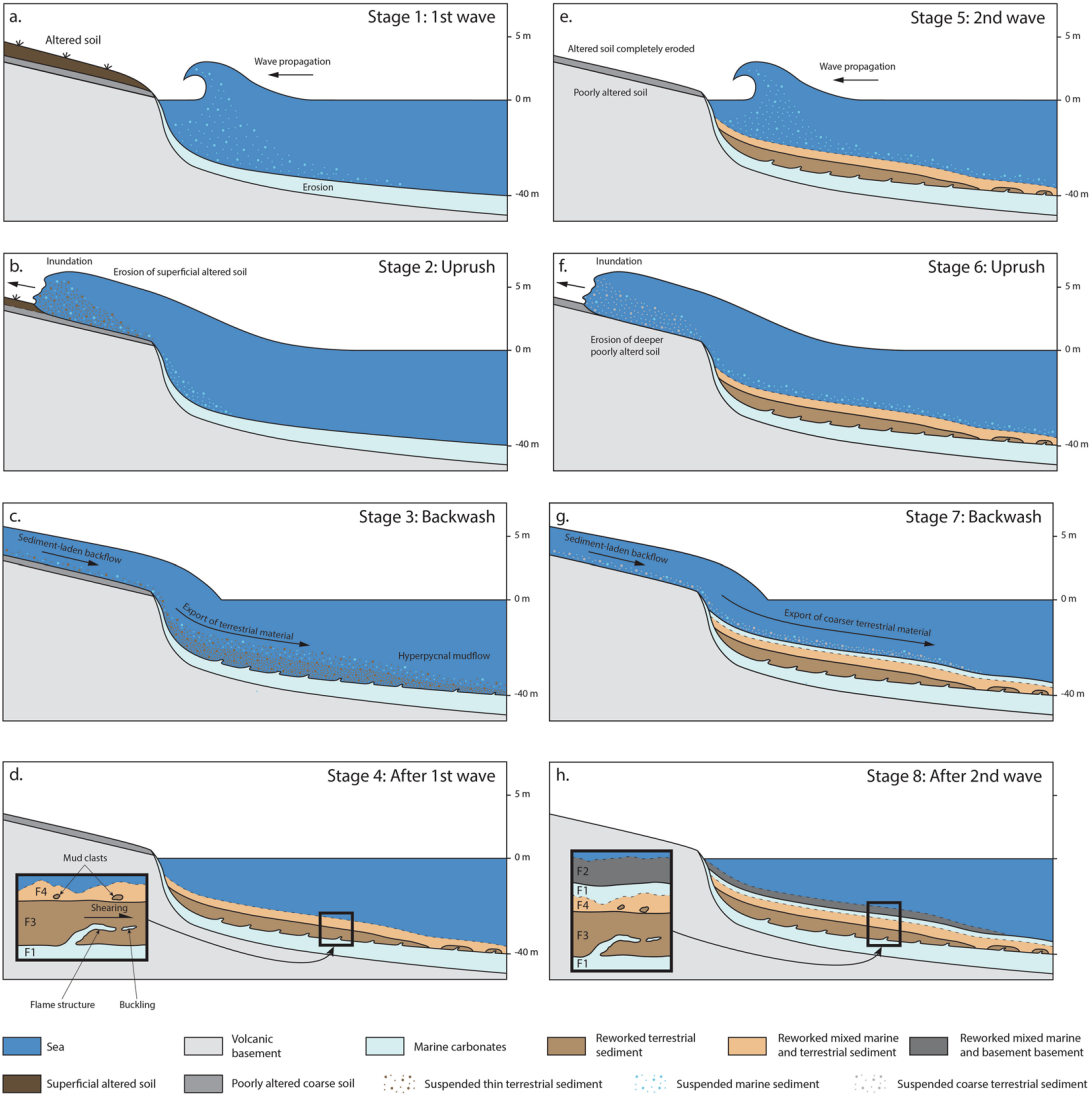
Interpretative tsunami backwash depositional model illustrating simplified transport and deposition mechanisms during each phase of the tsunami. The right-hand side panels represent the processes linked to a possible second wave such as during the 2009 SPT for the first hypothesis (Fig. 3a). The zoom in panel (d) illustrates a complete event layer in the case of a single major tsunami wave, such as seen in core CA08 for the 1960 GCET. The zoom in panel (h) illustrates a complete event layer in the case of two tsunami waves, such as seen in core CA08 for the 2009 SPT (first hypothesis, Fig. 3a).

The age model derived from short-lived radionuclides allowed to link the event layers to at least two specific events following both hypotheses. In the first hypothesis, the two shallowest Ti/Ca peaks in core CA08 (EL1, 4–6 cm and 8–10 cm, Fig. 3a), correlated with the shallowest peaks in all proximal cores (0–5 cm, CA02; 3–7 cm, CA09; 2–8 cm, CA07; 4–10 cm, CA08; 3–10 cm, CA04; 2–6 cm CA05, Fig. 4), are dated to AD 2009 ± 1 (EL1, Fig. 3a and 4). The deepest Ti/Ca peak in core CA08 (EL2, 40–42 cm, Fig. 3a), correlated with Ti/Ca increases at 48–50 cm depth in core CA04 and 47–49 cm depth in core CA09, (Fig. 4) are dated to AD 1960 ± 7 (EL2, Fig. 3a). Based on the age estimation of the two event layers, they are unlikely to have been caused by local flashfloods associated with the two major cyclones recorded in this period, Ofa (AD 1990) and Val (AD 1991). However, these two cyclones may be recorded by the small Ti/Ca peak at 24–26 cm depth in core CA08, dated between 1982 and 1992 (Fig. 3). Small dispersed clasts with similar mineralogical composition to facies F3 but showing no deformation structures are also observed at this depth (F5). The age estimations of event layers 1 and 2 based on ^210^Pb and ^137^Cs chronology match the date of two major historic tsunamis, with EL1 most likely to correspond to the 2009 SPT and EL2 to the 1960 GCET. In this case, the shallow peak observed in core CA08 (4–6 cm) may attest of the occurrence of two successive waves during the 2009 SPT (Fig. 7a to h). The first and second waves were 7 and 3 m high, respectively, compared to the single 3.5 m-high wave recorded for the 1960 GCET^22^. We hypothesize that the first wave eroded all the available superficial clay and the second wave eroded deeper into the coarser sediment. The thin F1-type sediment layer (1 cm) in-between facies F2 (above) and F3 and F4 (below) may be explained by older sediments reworked by the first wave and settled in between the two waves, or more likely marine sediment dragged from deeper parts of the bay by the uprush of the second wave and deposited on top of backwash sediments emplaced following the first wave (Fig. 7a to h). In this case, the absence of erosion and reworking of the sediment emplaced following the backwash of the first wave may be explained by the depth of the seafloor (34 m).

In the second hypothesis, the shallowest Ti/Ca peaks in core CA08 (EL1.1, 4–6 cm, Fig. 3b) is dated to AD 2009 ± 1 (EL1.1, Fig. 3b and 4). The second Ti/Ca peak (EL1.2, 7–10 cm, Fig. 3b), correlated with the shallowest peaks in all proximal cores (0–5 cm, CA02; 3–7 cm, CA09; 2–8 cm, CA07; 4–10 cm, CA08; 3–10 cm, CA04; 2–6 cm CA05, Fig. 4), is dated to AD 2008 ± 1 (EL1.2, Fig. 3b and 4). The deepest Ti/Ca peak in core CA08 (EL2, 40–42 cm, Fig. 3b), correlated with Ti/Ca increases at 48–50 cm depth in core CA04 and 47–49 cm depth in core CA09, (Fig. 4) are dated to AD 1959 ± 7 (EL 2, Fig. 3b). As for the first hypothesis, EL1.2 and EL2, which show similar sedimentological and geochemical characteristics, most likely correspond to the deposits emplaced following the 2009 SPT and the 1960 GCET, respectively (Fig. 7a to d). In this case, EL1.1, which is coarser than EL1.2 and EL2 but also characterized by a terrigenous origin, may attest of a localized run-off shortly after the 2009 SPT, provoked by a heavy rainfall event on slopes weakened by the impacts of the recent tsunami. Such heavy rainfalls could have been induced for instance by the February 12^th^ 2010 Tropical Cyclone Rene, just four months after the 2009 SPT. Indeed, several landslides were reported during this event^35,36^. Another possible candidate is Cyclone Wilma in January 2011, just over a year after the 2009 SPT, with heavy rainfall inducing several localized landslides^37^.

Flame structures such as those observed at the base of both event layers have occasionally been reported at the base of onshore tsunami uprush deposits, between successive waves^51^. In the context of submarine tsunami backwash, they have only been observed once by Le Roux *et al*.^9^ at greater scales and were attributed to large-scale mass failures induced by possible paleo-tsunamis. However, these observations are based on ancient deposits and are only inferred to have been caused by tsunami backwash with no independent proof of this specific event. Here, flame structures induced by shearing at the base of a hyperpycnal flow attributed to a tsunami backwash are observed for the first time in sediments emplaced by recent and well-documented historic tsunamis, and are proven to be of tsunami origin. Two reasons can be pointed out to explain why such micro-deformations have not been reported in earlier work. The first reason may be related to the specific morphology of the bay. Indeed, Pago Pago Bay is a deep and sheltered bay that may favor hyperpycnal flow during tsunami backwash leading to flame structures. The second reason could be that no one has yet analyzed thin sections in tsunami backwash deposits and thus have not been able to observe such features, despite their possible presence. Nevertheless, as this study proves, such sheltered environments provide an ideal preservation potential for event deposits and must not be neglected when searching for tsunami evidence.

Based on these observations, we propose the following sediment transport and deposition model for tsunami backwash based on hyperpycnal backflow (Fig. 7). During the uprush phase of the tsunami, the tsunami wave erodes and reworks the superficial onshore coastal sediment (Fig. 7a). During the backwash, a thin layer of water with highly concentrated reworked sediment is channelized and transported seaward and behaves as a hyperpycnal current after entering the sea (Fig. 7c). This results in the formation of a dense and cohesive inertia-driven mudflow similar to that described by Mulder *et al*.^52^ following the rupture of the Malpasset Dam. During this process, part of the superficial soft muddy sediment (F1) is incorporated due to shearing as rip-up clasts in the mudflow (F3), without mixing, in a buckling phenomenon. Finally, when the mudflow loses its inertia, it is deposited as a homogenous non- or poorly-graded layer (F3), topped by a thin transition layer (F4) due to the settling of suspended sediment (mixed F1 and F3) with a few mud clasts (F3) at the base (Fig. 7d).

## Conclusion

In this study, we present a unique 80-year sediment record in the sheltered Pago Pago Bay. Despite the absence of clear visual evidence in the cores, geochemical and thin section analyses combined with geochronological dating allowed the identification of backwash deposits of two major historic tsunamis, the 2009 South Pacific Tsunami and the 1960 Great Chilean Earthquake Tsunami. The geochemical analysis showed an increase of the Ti/Ca ratio in these layers, in line with the land-to-sea sediment transport observed around volcanic islands. Thin section analysis revealed the morphology of these deposits as a non- or poorly-graded layer exhibiting a sharp basal contact suggesting erosion. Micro-deformations revealed shearing and buckling structures indicating a gravity-driven and inertia-driven density current. These observations lead us to propose an improvement of the existing sediment transport and deposition model for tsunami backwash in sheltered environments, as a dense and cohesive hyperpycnal flow inducing shearing and buckling of the underlying superficial soft sediment. These observations represent a step further towards a better understanding of tsunami backwash flows. In addition, probable deposits induced by two recent tropical cyclones were identified and did not show basal micro-deformations. These deposits include potential fine-grained terrestrial flashflood deposits emplaced during Cyclone Ofa (AD 1990) or Val (AD 1991) and potential coarse-grained terrigenous run-off deposits emplaced during Cyclone Rene just four months after the 2009 SPT, or by Cyclone Wilma in early 2011. Thus, we propose a potential new proxy with the presence of basal shear and buckling micro-structures for the identification of tsunami backwash deposits in sheltered environments, which may also be used as a new criterion to differentiate tsunami and storm deposits. At a wider scale, this study has proven that sheltered bays offer an ideal preservation of event deposits and that it is urgent to consider such bays around the world for the reconstruction of paleo-tsunami catalogs and coastal risk assessment. Further work should concentrate on areas frequently impacted by tsunamis, such as the Pacific islands, Southeast Asia, Chile or even Alaska where deep and narrow sheltered calderas, bays or fjords could offer ideal sites for the preservation of tsunami deposits. Such studies could also validate the observations and interpretations provided in Pago Pago Bay and the basal micro-deformations as a tsunami proxy.

## Material and Methods

All data presented in this study were obtained during the oceanic campaign SAMOA-SPT from August 27^th^ to September 10^th^ 2015 aboard *R/V Alis*^21^. An extensive 2D high-resolution seismic survey was carried out, along with 1 m-resolution bathymetric and seafloor reflectivity surveys. A Seistec-IKB boomer was used for the seismic acquisition, with a vertical resolution of approximately 25 cm allowed by its 1 to 10 kHz bandwidth. All raw profiles were processed using iXBlue DELPH seismic acquisition software. A frequency filter was applied between 900 Hz and 10 500 Hz, coupled with a linear AGC (Amplitude Gain Control) and stacking of three adjacent traces. A Kongsberg EM-1002 multibeam sounder was used for the bathymetric and reflectivity acquisition with a 95 kHz frequency. Raw bathymetric data were processed and corrected for tide and salinity-induced celerity bias using IFREMER CARAIBES software, while the seafloor reflectivity was processed and corrected using IFREMER SonarScope software.

Ten short cores were sampled in Pago Pago Bay (Fig. 1) using a custom-made gravity box-coring device adapted from the CASQ (CAllypso SQuare) box corer. This coring device, with a maximum penetration of 30–60 cm, allows a slower and gentler penetration of the sediment surface, permitting an intact sampling of the superficial sediment record with preservation of sediment structures and laminae. Cores were sampled in water depth ranging from 27 to 47 m and their locations were chosen based on the raw seismic, bathymetric and reflectivity surveys. Cores were retrieved in topographic lows of the inner domain (Figs. 1 and 2), and in the outer domain, where the 2009 SPT backwash deposits were believed most likely to be found.

Cores were split, photographed and logged in detail, noting all physical sedimentary structures and the vertical facies succession. Grain size analysis of samples collected from all cores in 5 mm intervals was conducted using a Malvern Mastersizer S laser particle size analyzer. Statistical analysis of the grain size data was conducted using the Gradistat 8.0 software (Kenneth Pye Associates Ltd.). All cores were analysed for XRF using an Avaatech core scanner with a 1 mm measuring step. Two complementary runs were carried out for each core in order to count the full element spectrum: a first run at 10 kV and 1500 µA, and a second run at 30 kV and 2000 µA. XRF data was analyzed as element ratios and as separate elements normalized by the total counts. Thin sections were prepared for all short cores in 10 cm intervals. Smear samples were collected in two key areas of the watershed: two samples were collected onshore from Laolao and Pago streams (Fig. 1c), representing the terrestrial input into Pago Pago Bay, and two samples were collected on a beach (Fig. 1c), representing the bay sediments.

The chronology was established for core CA08 using 1-cm thick samples taken every 5 cm down to 57 cm depth (Fig. 1c). ^210^Pb, ^226^Ra and ^137^Cs activities were measured by gamma spectrometry at the Australian Nuclear Science and Technology Organisation (ANSTO, Lucas Heights, Australia). Approximately 3 g of samples were packed in 3 mL vials and sealed for 3 weeks before counting. Gamma photon peaks of ^210^Pb (46 keV), ^226^Ra (352 and 609 keV) and ^137^Cs (662 keV) were collected for more than 48 h using an Ortec well-type HPGe (High-Purity Germanium) detector. In each sample, the ^210^Pb excess activity (^210^Pb_ex_) was calculated by subtracting the ^226^Ra activity (the proxy for supported ^210^Pb) from the total ^210^Pb activity following Golberg (1963)^53^. The sedimentation rate was estimated using the CFCS (Constant Flux Constant Sedimentation) model^39^ and the age-to-death model was computed using serac R package^54^.
